# Retraction Note: The value of serum progesterone level on day of human chorionic gonadotrophin administration/metaphase II oocyte ratio in predicting IVF/ICSI outcome in patients with normal ovarian reserve

**DOI:** 10.1186/s13048-026-02302-8

**Published:** 2026-10-16

**Authors:** Ahmad Mahran, Mohammed Khairy, Reham Elkhateeb, Abdel Rahman Hegazy, Ayman Abdelmeged, Gaber El-Saber Batiha, Khalaf F. Alsharif, Helal F. Hetta, Haitham Ahmed Bahaa

**Affiliations:** 1 Department of Obsterics and Gynecology, Faculty of Medicine, Minia University, Minia, Egypt; 2 Department of Pharmacology and Therapeutics, Faculty of Veterinary Medicine, Damanhour University, Damanhour, Egypt; 3 Department of Clinical laboratory Sciences, College of Applied Medical Sciences, Taif University, Taif, Saudi Arabia; 4 Department of Medical Microbiology and Immunology, Faculty of Medicine, Assiut University, Assiut, Egypt

**Retraction Note: J Ovar Res 14, 52 (2021)**

**https://doi.org/10.1186/s13048-021-00800-5**

The Editors in Chief have retracted this article. Concerns were raised about the similarity between the ROC curves for P / E2 ratio and for serum P in Figs. 2 and 3. The authors did not respond to a request to provide their raw data. The Editors have therefore lost confidence in the integrity of the results reported in this article. The authors did not state explicitly whether they agree to this retraction.

